# In vitro function and in situ localization of Multidrug Resistance-associated Protein (MRP)1 (*ABCC1*) suggest a protective role against methyl mercury-induced oxidative stress in the human placenta

**DOI:** 10.1007/s00204-020-02900-5

**Published:** 2020-09-11

**Authors:** Sebastian Granitzer, Isabella Ellinger, Rumsha Khan, Katharina Gelles, Raimund Widhalm, Markus Hengstschläger, Harald Zeisler, Gernot Desoye, Lenka Tupova, Martina Ceckova, Hans Salzer, Claudia Gundacker

**Affiliations:** 1 Karl-Landsteiner Private University for Health Sciences, Krems, Austria; 2 Institute of Medical Genetics, Medical University of Vienna, Vienna, Austria; 3 Institute of Pathophysiology and Allergy Research, Medical University of Vienna, Vienna, Austria; 4 Department of Obstetrics and Gynecology, Medical University Vienna, Vienna, Austria; 5 Department of Obstetrics and Gynecology, Medical University of Graz, Graz, Austria; 6 Department of Pharmacology and Toxicology, Charles University, Hradec Kralove, Czech Republic; 7 Clinic for Pediatrics and Adolescent Medicine, University Hospital Tulln, Tulln, Austria

**Keywords:** Multidrug resistance-associated protein 1, Methyl mercury, HTR-8/SVneo, MDCKII, Oxidative stress, Human placenta

## Abstract

Methyl mercury (MeHg) is an organic highly toxic compound that is transported efficiently via the human placenta. Our previous data suggest that MeHg is taken up into placental cells by amino acid transporters while mercury export from placental cells mainly involves ATP binding cassette (ABC) transporters. We hypothesized that the ABC transporter multidrug resistance-associated protein (MRP)1 (*ABCC1*) plays an essential role in mercury export from the human placenta. Transwell transport studies with MRP1-overexpressing Madin-Darby Canine Kidney (MDCK)II cells confirmed the function of MRP1 in polarized mercury efflux. Consistent with this, siRNA-mediated MRP1 gene knockdown in the human placental cell line HTR-8/SVneo resulted in intracellular mercury accumulation, which was associated with reduced cell viability, accompanied by increased cytotoxicity, apoptosis, and oxidative stress as determined via the glutathione (GSH) status. In addition, the many sources claiming different localization of MRP1 in the placenta required a re-evaluation of its localization in placental tissue sections by immunofluorescence microscopy using an MRP1-specific antibody that was validated in-house. Taken together, our results show that (1) MRP1 preferentially mediates apical-to-basolateral mercury transport in epithelial cells, (2) MRP1 regulates the GSH status of placental cells, (3) MRP1 function has a decisive influence on the viability of placental cells exposed to low MeHg concentrations, and (4) the in situ localization of MRP1 corresponds to mercury transport from maternal circulation to the placenta and fetus. We conclude that MRP1 protects placental cells from MeHg-induced oxidative stress by exporting the toxic metal and by maintaining the placental cells' GSH status in equilibrium.

**Electronic supplementary material:**

The online version of this article (10.1007/s00204-020-02900-5) contains supplementary material, which is available to authorized users.

## Introduction

The metal mercury (Hg) is among the most hazardous chemicals with high significance for public health (WHO 2017, ATSDR 2019). The chemical species (elemental, inorganic or organic mercury), the dose and exposure duration determine its toxicity. Common cytotoxic characteristics of all forms of mercury are the disruption of the antioxidant system and the induction of apoptosis (Clarkson and Magos 2006; Yang et al. 2020). Mercury with its exceptionally high affinity to thiols is known to modify the redox state of its ligands. It affects the mitochondrial electron transfer chain leading to increased formation of reactive oxygen species (ROS), i.e. superoxide anion and hydrogen peroxide (Farina et al. 2013).

**Fig. 1:**
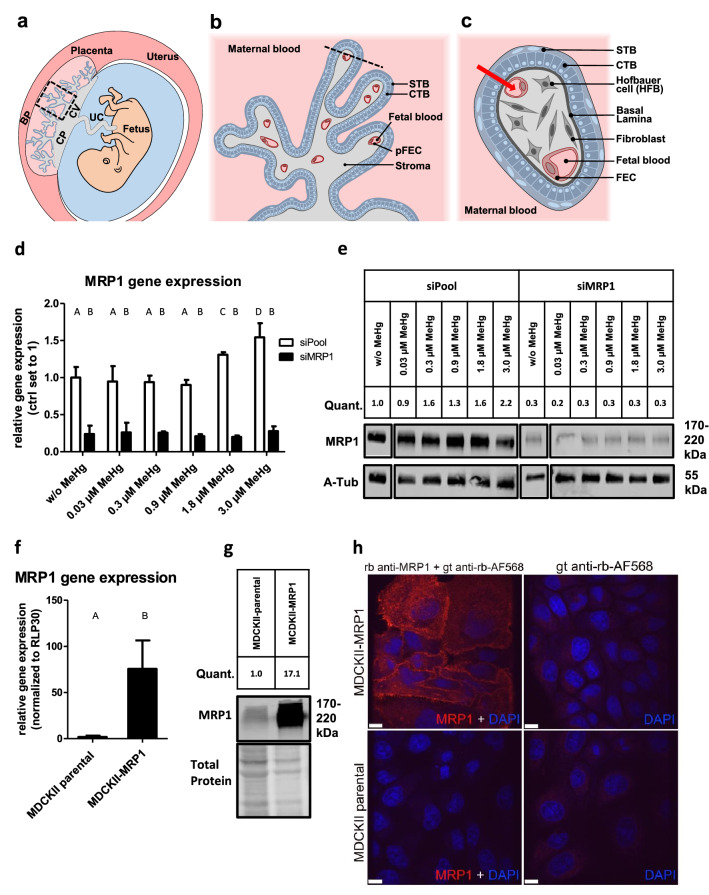
Structure of the human placenta and the placental barrier, validation of MRP1 expression levels in HTR-8/SVneo and MDCKII cells, and anti-MRP1 antibody specificity. **a** Position and structure of the human placenta including the chorionic plate (CP), the basal plate (BP) and the chorionic villi (CV). *UC* umbilical cord. **b** The early chorionic villus in a longitudinal section and **c** a cross section with trophoblast-derived structures (blue) and mesoderm-derived tissues (gray). The red arrow shows the path across the placental barrier, i.e. those cell layers (STB, CTB, pFEC) that a substance passes from maternal blood into fetal blood. As the CTB layer becomes discontinuous during pregnancy, in the late placenta there are only two cell layers (STB and pFEC) that a substance must cross. Slightly modified from Gundacker et al. (2016). **d** siRNA-mediated gene knockdown was performed in HTR-8/SVneo cells using MRP1-specific siRNA (siMRP1). Control cells were treated with non-targeting siRNA (siPool). In addition, cells were treated with or without (w/o) MeHg for 72 h. Gene knockdown was confirmed by RT-qPCR. **e** The anti-MRP1 antibody detected a protein of appropriate size (190 kDA) by western blotting in control cells, but hardly any in siMRP1 treated cells. **f** Relative human MRP1 gene expression levels of MDCKII cells constitutively expressing MRP1 and of MDCKII cells overexpressing MRP1 (MDCKII-MRP1) were analyzed by RT-qPCR. **g** Anti-MRP1 antibody detected a significant increase in protein expression in MDCKII-MRP1 cells (a representative western blot is shown). **h** In IFM, the anti-MRP1 antibody produced a strong fluorescence signal in MDCKII-MRP1 cells, but not in MDCKII cells or the negative controls. For quantification (quant.) of protein bands, MRP1 was normalized to either α-Tubulin (**e**) or Total Protein stain (**f**). RT-qPCR data represent mean values ± SD from 3 independent experiments, each performed in triplicates. The letters A-D denote homogeneous subgroups derived from one-way ANOVA and S–N-K posthoc test (*P* < 0.05)

The consumption of organic mercury (the most common form is methyl mercury—MeHg) through fish and shellfish is regarded as the main route of non-occupational exposure in humans (Sheehan et al. 2014). The fetal central nervous system is the primary target organ of MeHg toxicity. This was particularly evident in the cases of mass poisoning during the last century, when many children were born with severe neurological damage (Maccani et al. 2015). Mercury, in contrast to other heavy metals (e.g. cadmium), traverses the human placenta very efficiently (Gundacker and Hengstschlager 2012).The placental barrier, which is located in the placental chorionic villi (Fig. 1a), separates maternal from fetal blood circulation and enables the exchange of substances. It consists of an epithelial cell type, the syncytiotrophoblast (STB), which directly contacts maternal blood, the underlying cytotrophoblast (CTB) present as a continuous cell layer during early pregnancy, and the human placental fetal endothelial cells (pFECs) lining fetal blood vessels (Benirschke et al. 2012) (Fig. 1b, c). Overall, there is little data on how MeHg traverses these cell layers and how it affects cell functions when accumulating in placental cells (Straka et al. 2016; Tucker and Nowak 2018).

In various organs, cellular MeHg uptake is assumed to occur as a complex with cysteine that has structural similarity with methionine (Hoffmeyer et al. 2006), thereby involving amino acid transporters of the solute carrier (SLC) family (Bridges and Zalups 2017). To a certain extent, the efficient placental transmission of MeHg can be explained by its accidental uptake into trophoblast cells by methionine transporters LAT1 (SLC7A5) and LAT2 (SLC7A8) (Balthasar et al. 2017; Straka et al. 2016). Several studies have brought evidence for the assumption that most MeHg, when in a cell, quickly dissociates from cysteine (and therefore is no longer a substrate of amino acid transporters) to bind to the major antioxidant glutathione (GSH) (e.g. (Simmons-Willis et al. 2002) to be effluxed as GSH-conjugate by ATP-binding Cassette (ABC) transporters including the multidrug resistance associated protein 1 (MRP1; encoded by *ABCC1* gene) (Farina and Aschner 2019; Rush et al. 2012). MRP1 is not only the most important exporter of GSH-conjugates, and thus plays a key role in detoxification of cells from different xenobiotics (Cole and Deeley 2006) including mercury (Rush et al. 2012). The ability to export GSH and oxidized derivatives of GSH such as glutathione disulfide (GSSG), also endows MRP1 with the capacity to directly regulate the cellular thiol-redox status (Ballatori et al. 2009; Ellison and Richie 2012; Marchan et al. 2008).

Although our previous study suggested that MRP1 is involved in mercury efflux from human trophoblast cells (Straka et al. 2016), direct evidence was lacking. The main objective of the present study was thus to confirm the specific role of MRP1 in the transfer of MeHg from maternal to fetal blood circulation. First, we wanted to shed light on the role of MRP1 in the fetal-directed MeHg transport. ABC transporters can keep the harmful substances away from the fetal circulation (by active efflux from the apical membrane of the STB) or deliver molecules towards the fetal circulation depending on their expression and localization in the cell types of the placental barrier (Walker et al. 2017). We hypothesized that transepithelial transport of MeHg occurred predominantly in the apical-to-basal direction and studied involvement of MRP1 in vectorial MeHg transfer using Madin-Darby Canine Kidney (MDCK)II cells overexpressing human MRP1. Accordingly, we also expected higher amounts of mercury in MRP1-downregulated cells. We also hypothesized that MRP1 was not only important for placental cell detoxification, i.e. mercury excretion, but also for the antioxidant status of the cells. Thus, we examined effects of different MeHg concentrations on total Hg contents and GSH/GSSG status of the human trophoblast cell line HTR-8/SVneo in the absence and presence of MRP1 and evaluated MeHg cytotoxicity, cell viability, and apoptosis. MRP1 expression in human placenta is well established (Atkinson et al. 2003; Evseenko et al. 2006a, b; Pascolo et al. 2001; St-Pierre et al. 2000), but the in situ localization remains contradictory ranging from reports on sole or predominant STB localization (Afrouzian et al. 2018; Kozlowska-Rup et al. 2014) to expression in both STB and pFECs (Atkinson et al. 2003; Nagashige et al. 2003; St-Pierre et al. 2000). Moreover, the subcellular localization in the STB was unclear. Hence, our third aim was to address cellular and subcellular in situ localization of MRP1 in placental sections by immunofluorescence microscopy (IFM) using a validated antibody.

## Materials and methods

### Cell culture

HTR-8/SVneo cells (ATCC, CRL-3271™, Lot# 64275781) were cultured in RPMI-1640 medium (Gibco; 31870074), containing 5% fetal bovine serum (FBS; PanBiotech; P40-38100), 1% Glutamax (Gibco) and 1% Penicillin–Streptomycin-Neomycin Antibiotic Mixture (PSN; Gibco; 15640055). Cells were sub-cultured every 3–5 days. In experiments, culture medium without PSN was used. Cell number was determined with a CASY cell counter and analyzer (CASY; Innovatis Technologies Inc.).

MDCKII cells overexpressing human MRP1 and the relevant parental control cells were provided from Dr. A. Schinkel (Netherlands Cancer Institute, Amsterdam). Both lines were cultured in antibiotic-free high-glucose Dulbecco’s Modified Eagle Medium (DMEM) (Sigma Aldrich; D6429) supplemented with 10% FBS (Panbiotech; P40-37100). All the cells were cultured under 37 °C/5% CO_2_ conditions and periodically checked for *Mycoplasma* contamination (MycoAlert; Lonza). HTR-8/SVneo cells from passages 86 to 96 and MDCKII cells from passages 3–30 were used in the experiments.

Human placental endothelial cells (HPEC) as well as human trophoblast cells (hTC) used in immunoblot (Fig. 5) were isolated from healthy placentas (Department of Obstetrics and Gynecology, Medical University Vienna; EK 1035/2015; Department of Obstetrics and Gynecology, Medical University of Graz; EK 27-268 ex 14/15) according to previous studies (Lang et al. 2003; Straka et al. 2016).

### Methyl mercury (MeHg) dosages

MeHg (Alfa Aesar; 33.553.AC) was used in a concentration range from 0.03 µM, which is a non-cytotoxic dose (equivalent to about 6 µg/L) up to 3 µM (equivalent to 645 µg/L), which is a highly cytotoxic dose (Gundacker et al. 2011), also to primary trophoblast cells (Straka et al. 2016).

### GSH/GSSG assay

Total and oxidized GSH was quantified by GSH/GSSG Kit (Promega; V6611) according to the manufactures protocol. Reduced GSH was calculated according to the manufactures protocol (total GSH-oxidized GSH = reduced GSH). GSH measurement was normalized to cell number.

### Cytotoxicity and cell viability assays

Cell viability and cytotoxicity were measured simultaneously in a 96-well multiplex format. Cell viability was determined by RealTime-Glo MT Cell Viability Assay (Promega; G9711), while cytotoxicity was measured by CellTox Green Cytotoxicity Assay (Promega; G8741) according to the manufacturer’s protocol. In short, 1 × 10^3^ cells/well were seeded, on the next day treated with MeHg (0.03, 0.3, 0.9, 1.8, 3 µM) and analyzed at 2, 24, 48 and 72 h post treatment. Assay performance, i.e. reduction of cell viability and increase of cytotoxicity was controlled with 10 µM Ionomycin (Sigma; I9657-1MG) according to the manufacturer’s protocol (Suppl. Figure 1a and b).

### Apoptosis assay

Apoptosis was determined by Caspase-Glo 3/7 Assay System (Promega; G8090) in 96-well plates according to the manufacturer’s instructions. In short, 1 × 10^3^ cells/well were seeded, on the next day treated with MeHg (0.03, 0.3, 0.9, 1.8, 3 µM) and caspase activity was measured 48 h post treatment. 1 µM Staurosporine (Sigma; S5921-0.5MG) was used as positive control according to the manufacturer’s protocol (Suppl. Figure 1c).

### siRNA mediated knockdown

Cells were seeded in 6-well plates at a density of 1 × 10^5^ cells/well. On the next day, cells were transiently transfected with non-targeting siRNA (siPool = controls; GE Dharmacon; D-001810-10-20) and MRP1/*ABCC1*-specific siRNA (GE Dharmacon; L-007308-00-0020) using Lipofectamin RNAImax (Life Technologies; 13778075) as described previously (Balthasar et al. 2017; Rosner et al. 2010). Various siRNA concentrations were tested in combination with treatment of cells with 0.9 µM MeHg (see Suppl. Figure 2). A concentration of 50 nM was used in all subsequent experiments. Cells were cultured for 58 h and then MeHg (0.03, 0.3, 0.9, 1.8, 3 µM) was added for 72 h.

### Bi-directional transport of MeHg across monolayers of MDCKII-MRP1 and MDCKII-parental cells

MDCKII-MRP1 and MDCKII-parental cells were seeded on microporous polycarbonate mem-brane inserts (Costar; 3402; 3 mm pore size, 12 mm diameter) at a density of 5 × 10^5^ per insert and cultured for 72 h until the monolayer was formed as previously describe (Cihalova et al. 2015). The medium was replaced after 24 and 48 h of cultivation. The cells were then washed with pre-warmed phosphate-buffered saline (PBS) and preincubated in Opti-MEM with or without 50 µM MK-571 (MedChemExpress; L-660711), a MRP1 inhibitor (Tivnan et al. 2015) for 1 h, in order to address the contribution of MRP1 to MeHg transport. The transport assay was started by the addition of MeHg (0.2 µM) in Opti-MEM buffer to the apical (0.5 ml) or basolateral (1.5 ml) compartment of Transwell cell culture inserts. Samples of 50 µl were taken from the opposite (acceptor) compartments at timepoints 60, 120 and 240 min. At the end of the transport experiment, the solutions were removed from both compartments and cell monolayers rinsed 2-times with ice-cold (4 °C) PBS to stop the transporter activity. The microporous membranes were cut out from each insert and lysed in 0.02% sodium dodecyl sulfate (SDS) in ddH_2_O. Bi-directional transport data is presented as the percentage fraction of MeHg found in the acceptor compartments related to the initial stock solution applied to the donor compartment. The extent of MRP1-mediated transport is expressed as the efflux ratio (ER = Papp_A-B_/Papp_B-A_) relating permeable coefficients (Papp) of MeHg transport in the apical-to-basolateral (A-B) and basolateral-to-apical (B-A) directions within the linear phase of the transport assay.

### Protein extraction

Chorionic placental tissue samples were stored in RNALater (Thermo Scientific; AM7020) for at least 24 h at 4 °C. After 24 h, RNALater was discarded and placental tissue samples were stored at −20 °C until further processing. Placental tissue samples were processed with PARIS Kit (Thermo Scientific; AM1921) according to the manufacturer’s protocol. Protein concentrations were determined using Bradford reagent (BioRad; 500006)*.*

### Immunoblotting

Cells were lysed in RIPA Buffer (Thermo Scientific; 89901) supplemented with Halt Protease and Phosphatase Inhibitor Cocktail (Thermo Scientific; 78420) and 0.5 M EDTA solution (Thermo Scientific; 78430). 20 µg total protein was separated by 10% SDS-PAGE and transferred onto Odyssey Nitrocellulose Membranes (LI-COR). Membranes were dried for 10 min at 37 °C and then blocked for 1 h in Odyssey Blocking Buffer (tris-buffered saline: TBS) (LI-COR). Blots were incubated in TBS containing 0.1% Tween-20 (TBST) and the MRP1 (Cell Signaling; 72202; 1:1,000) and /or alpha tubulin (Merck; CP06; 1:1,000) primary antibody over night at 4 °C. On the following day, blots were washed with TBST and incubated with the secondary fluorophore-conjugated antibody (LI-COR; anti-mouse IR-Dye680; #92568070; 1:20,000/ anti-rabbit IR-Dye800; 92632211; 1:20,000) for 1 h at room temperature. The secondary antibodies were detected with the Odyssey CLx imager (LI-COR) using Image Studio Lite 5.2 software. REVERT™ Total Protein Stain (LI-COR) was used to detect total protein.

### RNA isolation, cDNA synthesis and quantitative PCR

RNA was isolated with TRI Reagent (Sigma; 93289-100ML) according to the manufacturer´s instructions. Total RNA was reverse transcribed using Go-Script Reverse Transcription System (Promega; A5001). cDNA was diluted 1:10 and 2 µl cDNA solution was used as template in gene expression assay reactions, following Applied Biosystems StepOnePlus Real-Time PCR System protocol. The employed primers were Hs00219905_m1 (*ABCC1*), Hs00824723_m1 (*UBC*) (Thermofisher) and 10041596 (*RLP30*) (BioRad).

### Analysis of total Hg

In vivo MeHg demethylation rate is very low (about 1% of body exposure per day) (Clarkson 2002). It must therefore be assumed that MeHg is practically not demethylated in in vitro experiments lasting a few days. Thus, the MeHg contents in supernatants and cells were analyzed as total Hg.

Cells, medium and reference material (Trace Elements Urine L-2, Lot 1403081) were digested with nitric acid (69%; Suprapur®; Roth; HN50.3) in a microwave oven (MARS6, CEM Corporation) and analyzed for total Hg by atomic fluorescence spectroscopy (mercur plus, Analytic Jena). The concentrations of the reference material (42.4 ± 0.98; *n* = 3; Recovery 96 ± 2%) lay well within the certified range (Hg: 44.0 µg/L, range: 35.2–52.9 µg/L). The limit of detection was 0.012 µg/L (*n* = 3). All samples were measured in duplicate (RSD < 1%) in the appropriate dilution and concentrations were calculated from a standard curve (0.0–3.2 µg/L).

### Immunofluorescence microscopy (IFM)

Human placentas (*n* = 5) were obtained within 15 min after cesarean sections of healthy pregnancies at 38–40 weeks of gestation (Department of Obstetrics and Gynecology, Medical University Vienna; EK 1035/2015). The tissues were transferred to the laboratory at room temperature within 15 min. For IFM, chorionic tissue was immediately processed by HOPE-fixation (DCS Innovative Diagnostik-Systeme) and paraffin-embedding (Blaschitz et al. 2008). Tissue sections (4 µm) were de-waxed and rehydrated (Blaschitz et al. 2000). Antigen retrieval was done with 0.05% (v/v) citraconic anhydride solution, pH 7.4, for 20 min (Leong and Haffajee 2010). Sections were incubated with blocking buffer (5% (v/v) goat serum (Jackson ImmunoResearch Laboratories; 005-000-121) in PBS containing 0.05% (w/v) saponin (Sigma; SAE0073) for 1 h at room temperature. Primary antibodies and corresponding Alexa-Fluor®-conjugated secondary antibodies (Table 1), diluted in blocking buffer, were applied overnight at 4 °C or for 2 h at room temperature, respectively. In negative control incubations, primary antibodies were omitted. Nuclei were stained with 4′,6-diamidino-2-phenylindole, dihydrochloride (DAPI; Roche Diagnostics GmbH; 10236276001; 50 µg/mL in PBS). After each incubation step, sections were washed intensively with PBS. In co-localization studies, multiplex staining was done where antibodies were added in the following order: rb anti-hMRP1, gt anti-rb IgG-AF647, m anti-CK7, gt anti-m-IgG AF488, m anti-CD31, gt anti-m IgG AF568, DAPI. Fluoromount-G (SouthernBiotech; 0100-01) was used as mounting medium. Images were acquired using an automated widefield fluorescence microscope (Axio Imager Z1, Zeiss), equipped with an EC Plan-Neofluar 20x/0.5 objective (Plan-Neofluar, Zeiss) and the following filter sets (Chroma Technology Corp.): 49000 ET-DAPI, 49002 ET-FITC/Cy2, 49008 ET-mCherry, TxRed, and 49006 ET-Cy5 in combination with TissueFAXS Image Acquisition and Management Software (Version 6.0; TissueGnostics GmbH). Using a monochrome camera (Hamamatsu), grayscale images of individual fluorescence channels were acquired. Acquired regions were composed of at least 5 × 5 single images. Pseudo-colors were assigned to the individual images and selected fluorescence channels were combined and exported when appropriate.

**Table 1:** Antibodies used in immunofluorescence microscopy

Antigen	Company	Host	Dilution Conjugated fluorochrome
Human MRP1	Cell Signaling 72202	Rabbit	1:50 placenta; 1:200 MDCKII cells
Rabbit IgG	Thermo Fisher Scientific A-21244	Goat	1:2,000 Alexa-Fluor-647
Human CK7	Agilent Dako M7018	Mouse	1:200
Mouse IgG	Thermo Fisher Scientific A-11001	Goat	1:2,000 Alexa-Fluor-488
Human CD31	Agilent Dako M0823	Mouse	1:100
Mouse IgG	Thermo Fisher Scientific A-11004	Goat	1:2,000 Alexa-Fluor-568

Alternatively, confocal images were acquired using an UltraVIEW ERS Rapid Confocal Imager (Perkin-Elmer) connected to a Zeiss Axiovert 200 microscope fitted with a 63x/1.4 oil objective lens (Plan-Apochromat, Zeiss). The fluorophores were excited using a 488/548/647 multiline argon/krypton laser. Pictures were digitalized and processed by Volocity software (Version 5.5, Perkin Elmer). Individual fluorescence channels displayed in pseudo-colors were combined and exported. Representative images were further processed with Adobe Photoshop CS5 Version 12.0.4 using identical conditions for positive and negative controls (Suppl. Figure 3).

MDCKII-parental and MDCKII-MRP1 cells were seeded in 8-well chamber slides (Ibidi; 80826). The next day, cells were fixed with Image-IT™ Fixative Solution (Thermo Scientific; R37814) containing 4% (v/v) of formaldehyde for 15 min. All subsequent incubations were performed at room temperature. Cells were washed with PBS and incubated with blocking buffer for 30 min. The primary anti-human MRP1 antibody (Table 1) was diluted 1:200 in blocking buffer and added to cells for 2 h. Blocking buffer without primary antibody was added to the negative control cells. After washing with PBS (3 × 5 min), an Alexa Fluor-568-labeled secondary antibody (diluted 1:2,000 in blocking buffer; Table 1) was allowed to bind to the cells for 1 h. Chambers were washed 3 × 5 min with PBS. For staining of nuclei, DRAQ5 (Thermo Scientific, 62251) diluted 1:200 in PBS was added for 10 min, followed by washing with PBS. Thereafter, staining was evaluated by confocal microscopy.

### Statistics

Data were obtained from at least 3 independent experiments (3 passages) made in triplicate and represent mean values ± standard deviation (SD). One-way ANOVA was used for statistical analysis of group differences, followed by a student–Newman–Keuls (S–N–K) test to correct for multiple testing (homogeneous subgroups are labeled with the same letters). Parametric student´s t-test was applied for statistical analysis of transport experiments. Calculations were performed via IBM SPSS25 and charts were created in GraphPad Prism 6 software. The significance level was set to *α* = 0.05.

## Results

### Specificity of the anti-MRP1 antibody and MRP1 expression in HTR-8/SVneo cells and MDCKII cells

For the validation of a rabbit monoclonal antibody from Cell Signaling (MRP1/ABCC1 (D5C1X) Rabbit mAb 72202), recommended for use in western blotting, immunohistochemistry (IHC) and immunoprecipitation, we followed the recommendations of Bordeaux et al. (2010). We performed western blotting with total tissue lysates as in IFM/IHC the antibody is also used on total tissue. The antibody has been produced by immunizing animals with a synthetic peptide corresponding to residues surrounding Val273. We analyzed sequence similarity of this area, which is located in the third cytoplasmic loop of MRP1, with the other MRP transporters described to be expressed in human placenta (MRP2-MRP5) (Dallmann et al. 2019). A protein BLAST search to align this sequence with human MRP2-5 found 36, 41, 30, and 30% homology, respectively. The company specifies in the datasheet that the antibody does not cross react with MRP2 or MRP3.

In HTR-8/SVneo cells (without MRP1 knockdown), treatment with increasing MeHg concentrations induced a dose-dependent rise in gene and protein expression of MRP1. Upon siRNA-mediated gene knockdown of MRP1, we observed a significant decrease of MRP1 expression in HTR-8/SVneo (Fig. 1d, e) and HeLa cells (data for the latter not shown). The overexpression of MRP1 in the MDCKII-MRP1 cell line compared to the parental cells (MDCKII-parental) was confirmed at the gene expression level (Fig. 1f). The anti-MRP1 antibody also detected significantly more protein in MDCKII-MRP1 cells than in MDCKII-parental cells, both in immunoblotting (Fig. 1g) and IFM (Fig. 1h). The strong fluorescence signal in the transfected cells indicates that the antibody also reacts with MRP1 protein after chemical fixation. The specificity of the anti-MRP1 antibody was thus confirmed for both applications.

### Preferential MeHg transport in the apical-to-basolateral direction

**Fig. 2:**
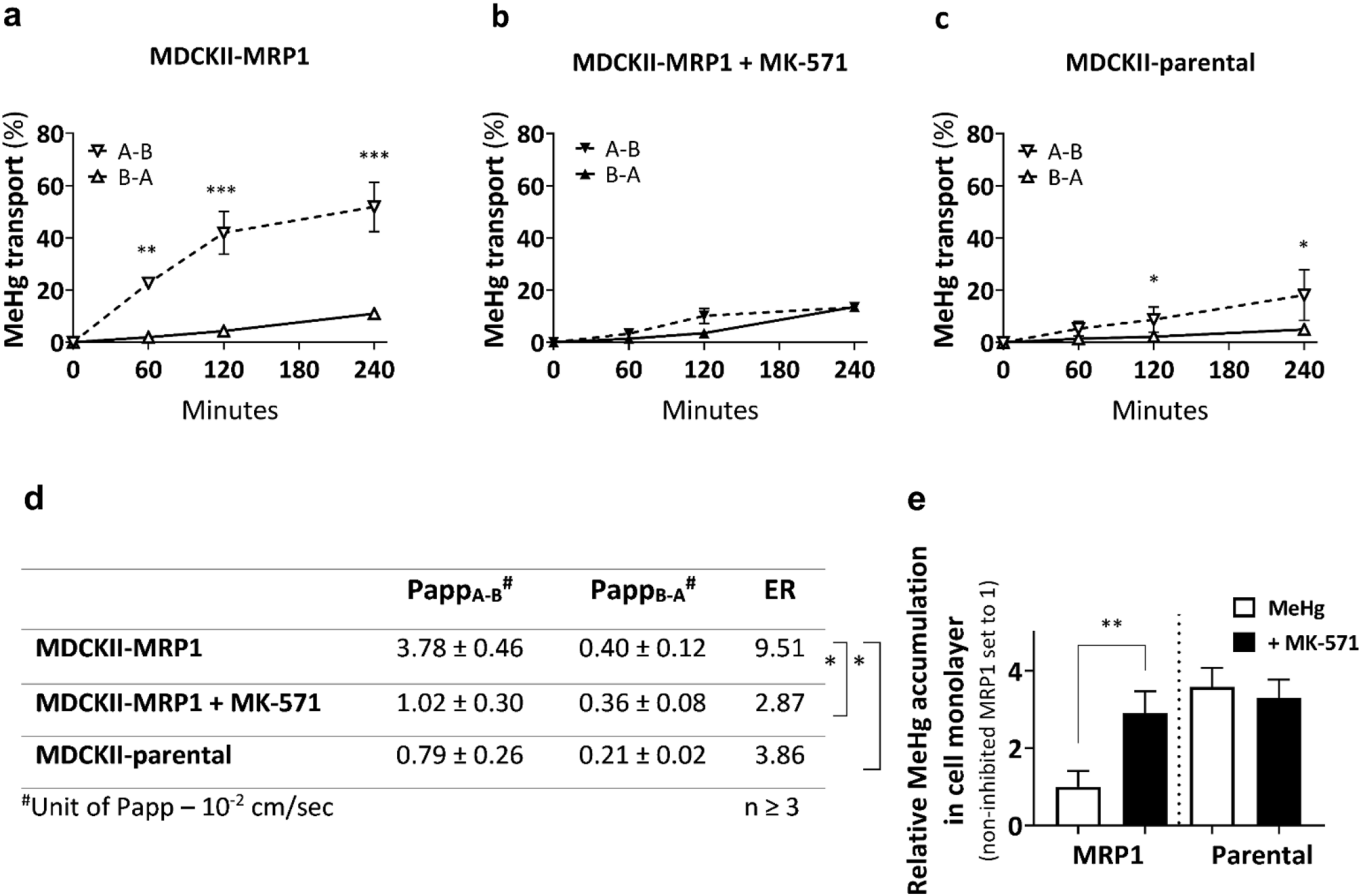
MeHg transport across MDCKII-parental and MDCKII-MRP1 cells over 4 h. **a** Predominant transport of MeHg in the apical-to-basolateral (A–B) direction in MDCKII-MRP1 cells. **b** MRP1 inhibitor MK-571 (50 µM) decreased A–B MeHg transport in MDCKII-MRP1 cells. **c** MDCKII-parental cells show reduced A–B MeHg transport than MDCKII-MRP1 cells. **d** Values of permeable coefficient (Papp) were calculated at 120 min within linear phase of MeHg transport. Statistical significance between efflux ratios (ER = Papp_A-B_/Papp_B-A_) of MeHg transport in MDCKII-MRP1 (ER = 9.51), MDCKII-MRP1 + MK-571 (ER = 2.87) and MDCKII-parental cells (ER = 3.86) revealed MeHg as MRP1 substrate. **e** Retention of MeHg in cell monolayer after transport assay. Data are presented as means ± SD (*n* ≥ 3). Statistical significance was evaluated by parametric student’s *t*-test (*P ≤ 0.05, **P ≤ 0.01, ***P ≤ 0.001)

The transport across monolayers of MDCKII-MRP1 that overexpress human MRP1 mainly on the basolateral plasma membrane (Evers et al. 2000) revealed a preferential transport of MeHg in the apical-to-basolateral (A–B) direction in comparison to MeHg transport in the opposite, basolateral-to-apical (B–A) direction (Fig. 2a). Addition of the MRP1 transporter inhibitor MK-571 reduced the efflux of total Hg (Fig. 2b), diminishing thereby the efflux ratio (ER) from 9.51 to 2.87 (Fig. 2d) and confirming MeHg as a substrate of the human MRP1 transporter. Only small asymmetry was observed in the MeHg transport across the MDCKII-parental cells (Fig. 2c) resulting in ER 3.86 (Fig. 2d) and suggesting involvement of an endogenous canine Mrp transporter. Addition of MK-571 to the MDCKII-parental cells further decreased this ratio to 1.90 (data not shown). When having analyzed all cellular monolayers at the end of the transport experiment, we observed the lowest accumulation of total Hg inside the MDCKII-MRP1 monolayers, which increased almost 3 times in the presence of MK-571, reaching thereby the total Hg levels found in the MDCKII-parental cells (Fig. 2e).

### MRP1 downregulation of MeHg-treated HTR-8/SVneo cells increases cellular mercury content, cytotoxicity and apoptosis, and decreases cell viability

**Fig. 3:**
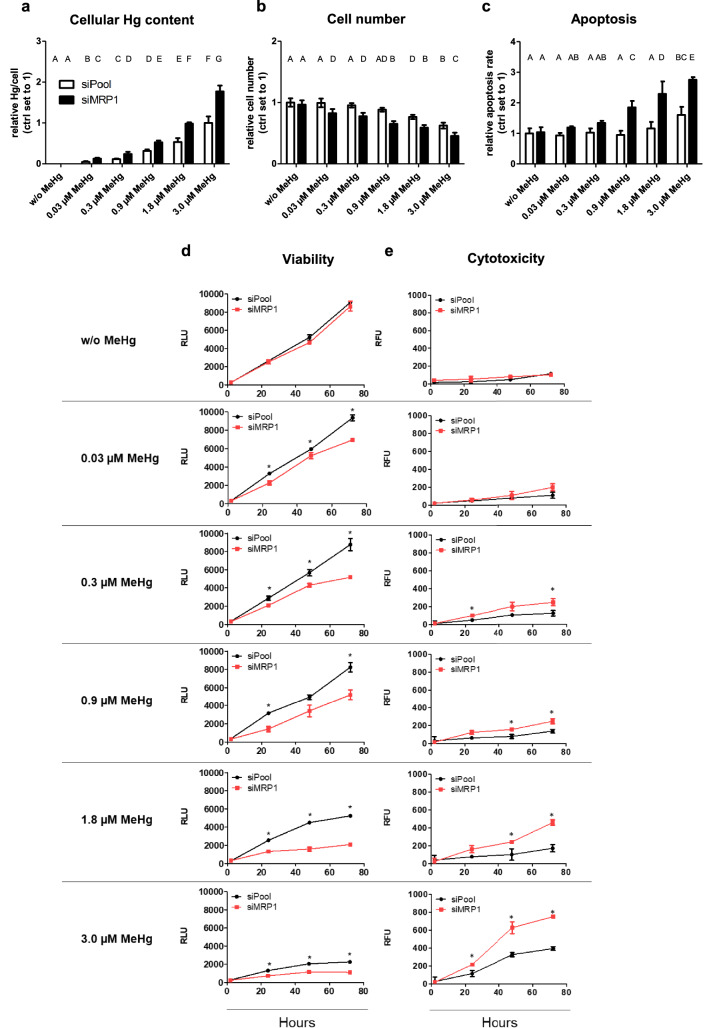
Effects of MeHg treatment and MRP1 downregulation on total Hg content, cell number, apoptosis, viability and cytotoxicity in HTR-8/SVneo cells. In a dose-dependent manner, MeHg exposure caused **a** an increase in cellular mercury content, **b** a decrease of cell number and **c** an increase of apoptosis relative to controls (w/o MeHg). In relation to controls (siPool) cell viability decreased (**d**) and cytotoxicity increased (**e**) after MeHg treatment in a dose-dependent manner. MRP1 knockdown intensified these effects. RLU: Relative Luminescence Unit; RFU: Relative Fluorescence Unit. The data represent mean values ± SD from three independent experiments, each performed in triplicates. **a–c** The letters A–G denote homogeneous subgroups derived from one-way ANOVA and S–N-K posthoc test (*P* < 0.05). **d**, **e** Asterisks denote significant differences between controls and MRP1 downregulated cells, *P < 0.05 from students t-test

Exposure of HTR-8/SVneo cells to increasing MeHg concentrations over 72 h leads to elevated cellular Hg levels. In MRP1 knockdown cells, the total Hg content was almost twice as high as in control cells (Fig. 3a). MeHg treatment reduced the cell numbers in a dose-dependent manner. This effect was further enhanced by the knockdown of MRP1, where already the concentration of the lowest MeHg dose (0.03 µM) significantly reduced the cell count (Fig. 3b). In control cells, apoptosis was induced at 3.0 µM MeHg, whereas after MRP1 depletion an increase in apoptosis was observed already at 0.03 µM MeHg (Fig. 3c). Without MeHg, cell viability was not affected by MRP1 knockdown over a period of 72 h (Fig. 3d). Accordingly, no cytotoxic effects were observed (Fig. 3e). Control cell viability was not affected at lower MeHg doses but was significantly reduced at concentrations of 1.8 and 3.0 µM MeHg. The MRP1 knockdown reduced cell viability even at the lowest dose (0.03 µM MeHg) (Fig. 3d). Similarly, cytotoxicity in control cells occurred only at the higher concentrations of 1.8 and 3.0 µM MeHg (Fig. 3e). In MRP1 silenced cells MeHg doses ≥ 0.3 µM showed significant cytotoxicity.

### MRP1 knockdown impairs the GSH status of MeHg-treated HTR-8/SVneo cells

**Fig. 4:**
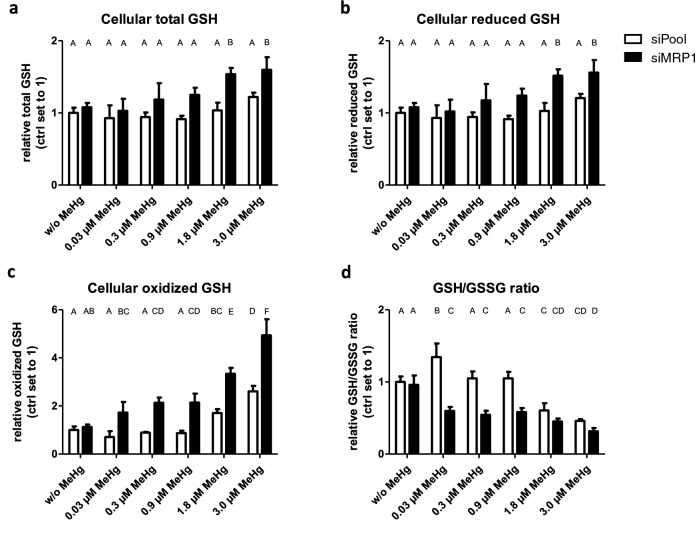
Effects of MeHg treatment and MRP1 downregulation on GSH status in HTR-8/SVneo cells. Exposure to MeHg led to dose-dependent increases in total GSH (**a**), reduced GSH (**b**) and oxidized GSH (**c**) and decreased the GSH/GSSG ratio (**d**) relative to the controls (w/o MeHg). The MRP1 knockdown enhanced these effects. The data represent mean values ± SD of three independent experiments, each performed in triplicates. The letters A–F denote homogeneous subgroups derived from one-way ANOVA and S–N–K post-hoc test (*P* < 0.05)

Without MeHg treatment, the MRP1 knockdown had no effect on the GSH/GSSG ratio (Fig. 4a–d).

In control cells with constitutive MRP1 expression, only high MeHg doses (1.8 and 3.0 µM) increased the levels of oxidized GSH (GSSG) (Fig. 4c). MeHg treatment resulted in a non-monotonic response in the GSH/GSSG ratio, with a significant increase at the lowest dose of 0.03 µM MeHg (indicating less oxidative stress than in untreated controls), no change at intermediate doses (0.3 and 0.9 µM), and an abrupt decrease at higher doses (1.8 and 3 µM) indicating severe oxidative stress (Fig. 4d).

In MRP1 knockdown cells, the increase in total GSH, reduced GSH, and GSSG was more pronounced and was already observed at 0.03 µM MeHg for GSSG (Fig. 4a–c). Likewise, a significant drop in the GSH/GSSG ratio was already evident at 0.03 µM MeHg (Fig. 4d). It has to be noted that the effect of MRP1 knockdown on GSH status is only detected in MeHg-treated cells. This means that MRP1 only affects the cellular GSH status or the GSH/GSSG ratio if MeHg (even in small amounts) is present.

### MRP1 is expressed in STB and pFECs in situ

**Fig. 5:**
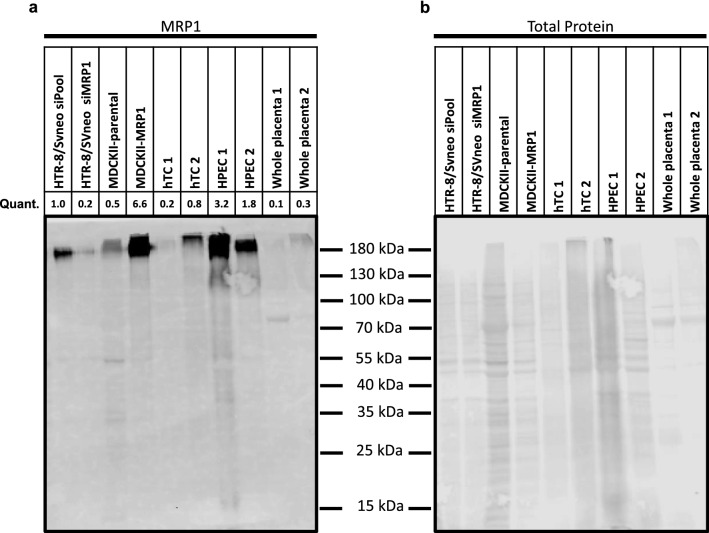
Western blot analysis of MRP1 expression in HTR-8/SVneo and MDCKII cells in comparison to placenta-derived primary cells and term placental chorionic tissue. **a** Western blot assay for the expression of MRP1. **b** Relative protein band intensity in REVERT™ Total Protein Stain. In the case of primary cells (hTC, HPEC) and whole placental tissue, samples from two placentas each were used. The quantification (MRP1 was normalized to Total Protein stain) is based on the Western blot shown. hTC: human primary trophoblast cells; HPEC: human placental endothelial cells

By western blotting, anti-MRP1 antibody detected a molecule of the appropriate size [190 kDa; (Cole et al. 1992)] in whole placenta lysates as well as in lysates of isolated human trophoblasts (hTCs) and human placental endothelial cells (HPEC) (Fig. 5a; Total protein staining shown in Fig. 5b). The highest constitutive expression level was observed in HPEC (Fig. 5a). Lysates of HTR-8/SVneo cells with siRNA-mediated gene knockdown (siMRP1) and of control cells (siPool) as well as MDCKII-parental and MDCKII-MRP1 cells were included as controls. For the use of the anti-MRP1 antibody in IFM on placental tissue, we tested various antibody dilutions (1:50–1:500, data not shown). While a strong placental endothelial cell staining remained visible at any antibody dilution, staining of the STB got lost upon higher dilution of the antibody. Due to the result of the western blots, we are confident that placental trophoblast cells express MRP1 and thus, we used an antibody dilution of 1:50 in all subsequent localization experiments.

**Fig. 6:**
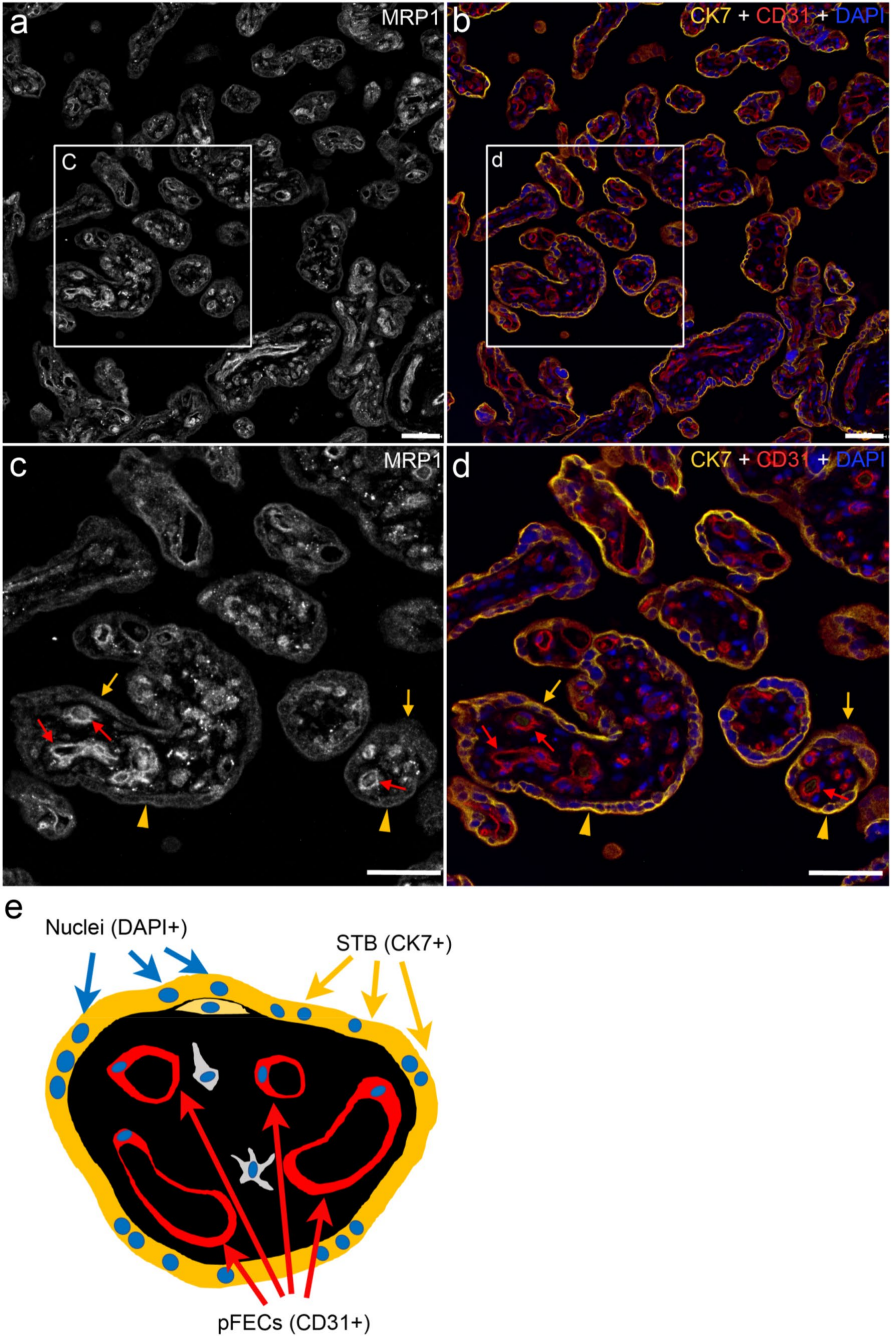
Localization of MRP1 in third trimester placental chorionic tissue analyzed by multiplex staining and widefield fluorescence microscopy. MRP1 expression (white in **a** and **c**) was observed in the STB (yellow arrows and arrowheads) and pFECs (red arrows) of all villi. STB and pFECs were identified by CK7 (yellow) and CD31 (red) expression, respectively (**b**, **d**). Nuclei were stained with DAPI (blue, **b**, **d**). A cartoon depicting the location of STB and pFECs in a term chorionic villus is shown in **e**. Within the STB, MRP1 staining appeared as vesicular pattern (yellow arrows) as well as membrane lining (yellow arrowheads). Representative images of one out of five analyzed placentas are displayed. Images were taken with a 20 × objective. **c** and **d** Represent enlarged areas of **a** and **b**, respectively. Bars represent 50 µm (color figure online)

To verify localization of MRP1 in the CK7-positive STB as well as CD31-positive pFECs, a multiplex IFM was performed, which enabled simultaneous detection of MRP1, CK7 and CD31 by specific primary and corresponding fluorescence-conjugated secondary antibodies. We display MRP1 staining in white, while in corresponding areas the cell markers CK7 (yellow) and CD31 (red) as well as DAPI (labeling nuclei in blue) are shown in merged images. A cartoon of a cross section of a chorionic villus is depicted in Fig. 6e, where STB, pFECs and nuclei are color-coded identical to the IFM images displaying these structures.

Analysis of healthy chorionic tissues by widefield immunofluorescence microscopy (Fig. 6) revealed MRP1 expression (Fig. 6a and magnified in Fig. 6c) in the CK7-positive STB (yellow arrows and arrowheads in Fig. 6c, d) as well as CD31-positive pFECs (red arrows in Fig. 6c, d). In analogy to the immunoblot (Fig. 5), the MRP1 signal was brighter in pFECs than in the STB, confirming higher expression levels in pFECs. The subcellular localization of MRP1 within the STB varied, ranging from vesicular intracellular staining (yellow arrows in Fig. 6c, d) to plasma membrane localization (yellow arrowheads in Fig. 6c, d).

The experiments for MRP1 localization in human healthy chorionic tissue were performed with 5 different placentas with at least 3 technical repetitions per individual placenta. Two different batches of the antibody were used. Identical results were observed in all cases (Compare Fig. 6 and Suppl. Figure 3, where the results from 2 other placentas are displayed).

**Fig. 7:**
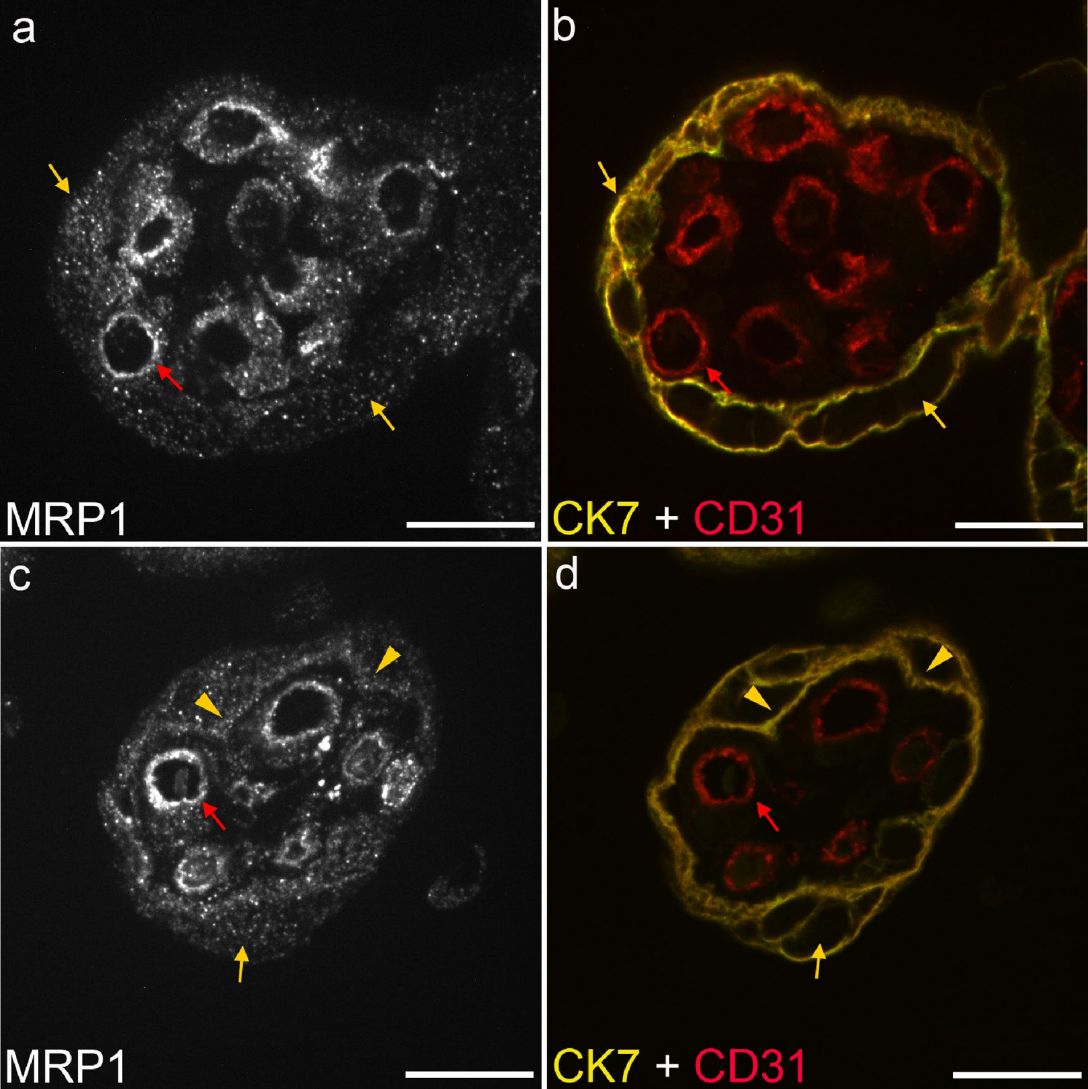
Localization of MRP1 in third trimester placental chorionic villi analyzed by multiplex staining and confocal microscopy. MRP1 expression (white in **a** and **c**) was observed in the STB (yellow arrows and arrowheads) and pFECs (red arrows) of the villi. STB and pFECs were identified by CK7 (yellow) and CD31 (red) expression, respectively (**b**, **d**). Within the STB, MRP1 was observed as vesicular pattern (yellow arrows) as well as basal membrane lining (yellow arrowheads). In the pFECs, MRP1 staining appeared vesicular (red arrows) with occasional enrichment at the abluminal and luminal cell surface. Representative images of two out of five analyzed placentas are displayed. Images were taken with a 63 × objective. Bars represent 20 µm (color figure online)

To reduce secondary fluorescence obscuring resolution of features in the focal plane and thus better reveal the subcellular localization of MRP1, we also collected optical sections from the IFM-stained samples by confocal microscopy. Two examples of chorionic villi are displayed in Fig. 7 (For orientation, compare to the cartoon in Fig. 6e). In the STB (labeled via CK7 in Fig. 7b, d, yellow), we observed MRP1 localization often in intracellular vesicles (Fig. 7a, c, arrows). In some, but not all villi, localization at (or close to) the basal membrane of the STB was found (Fig. 7b, arrowheads). The basal membrane of the STB is directed towards the stroma of the villi. The pFECs (labeled via CD31 in Fig. 7b, d, red arrows) also showed a vesicular intracellular localization of MRP1 with variable enrichment on the abluminal and/or luminal surface of the cells.

## Discussion

### Transepithelial transport of mercury through MRP1

Transport assays employing monolayers of MDCKII-MRP1 cells clearly demonstrated human MRP1-driven transfer of MeHg in apical-to-basolateral direction. According to the decision tree for *ABCB1 and ABCG2* transporter substrates introduced by the International Transporter Consortium, a compound is considered as a substrate of the tested transporters if its net flux ratio is ≥ 2 (i.e., the efflux ratio in the transporter-overexpressing system is at least twice as high than in the control one) and when a model inhibitor reduces the transport of the substrate by at least 50% in the transporter-overexpressing system (Giacomini et al. 2010). Applied to the MDCKII-MRP1 monolayers, the efflux ratio reached 9.51 compared to 3.86 in the MDCKII-parental cells, resulting in the net flux ratio of 2.46 clearly exceeding the cut-off value and confirming MeHg as the MRP1 substrate. Moreover, the model inhibitor diminished the transport by more than 3 times. A slight asymmetry in MeHg transfer between MDCKII parent cells, which favors the involvement of the endogenous Mrp transporter, most likely Mrp1, in apical-basolateral transfer, has been shown for other substrates in MDCKII wild-type cells (Zhao et al. 2019). This is in line with the fact that our anti-MRP1 antibody detected a faint band in MDCKII-parental cells (Fig. 1d). The algorithm BLASTP (NIH) revealed > 80% sequence homology between the third cytoplasmic loop of human MRP1 (the binding site of the antibody) and *Canis lupus familiaris*, which explains reactivity of the antibody with both, human and canine MRP1. The anti-MRP1 staining pattern we observed upon IFM further confirms this finding. Considering the MDCKII cells as a model of polarized epithelia arising from canine renal tubules, the direction of MeHg permeability across this monolayer fully correlates with the older experiment demonstrating rapid transport of MeHg across the tubular epithelium of the perfused proximal tubule in rabbits (Zalups and Barfuss 1993). Diminished intracellular levels of MeHg observed at the end of our transport experiments further indicate the active role of MRP1 in the clearance of MeHg from the cells, ensuring their protection.

### The effect of MeHg on HTR-8/SVneo cells in absence and presence of MRP1

In accordance with previous reports (Biondi et al. 2010; Parry and Zhang 2007), we show that HTR-8/SVneo, an immortalized first-trimester human trophoblast cell line, expresses MRP1. HTR-8/SVneo cells respond to MeHg treatment with a dose-dependent upregulation of MRP1 expression confirming MRP1 is required in tissue detoxification (Long et al. 2011). The elevated rates of apoptosis, membrane leakage and reduced metabolic activity in HTR-8/SVneo cells at higher MeHg concentrations (≥ 0.9 µM) that we have observed are in line with a recent report, where concentrations above 0.5 µM MeHg also impaired cell proliferation of HTR-8/SVneo cells (Tucker and Nowak 2018). However, after MRP1 depletion, these effects were observed already at the lowest MeHg dose (0.03 µM), which is in the range of a physiological MeHg (6 µg/L) concentration. The finding shows for the first time the crucial role of MRP1 in protecting cells from MeHg-induced cytotoxicity. This may also apply to other cells and to a number of other endogenous and exogenous toxic metabolites that are MRP1 substrates.

The mechanism behind the protective role of MRP1 may rely in its basal as well as apoptotic release of various glutathione forms (GSH, GSSG, GS-X) (Cole and Deeley 2006). In cultured astrocytes, it has been shown that MRP1 mediates 60% of the GSH export and is exclusively responsible for GSSG export (Minich et al. 2006; Steinmeier and Dringen 2019). We observed a significant reduction in the GSH-GSSG ratio indicating a strong imbalance in the amounts of reduced to oxidized GSH in MeHg-treated HTR-8/SVneo cells. Also, this adverse effect was further enhanced in the absence of MRP1. As a result, MeHg accumulates in the cells (clearly indicated by the increase in cellular total Hg levels) at a level that may exceed the binding capacity by cellular thiols. The shift we observed in the GSH-GSSG ratio towards more total GSH (indicating increased de novo synthesis)—but in particular towards the oxidized form (GSSG), which increases by a multiple—shows that relatively less reduced glutathione (GSH) is available to counteract the pro-oxidative properties of MeHg. This may lead to the production of ROS that damage lipids, proteins and nucleic acids and eventually lead to cell death (Farina et al. 2011).

The individual susceptibility to mercury toxicity is not merely dependent on MRP1, but determined by a complex network of proteins involved in binding (metallothioneins, selenoproteins), detoxification (glutathione system), and transport (xenobiotic transport proteins) of the metal (Andreoli and Sprovieri 2017). Nevertheless, our results show that even very low doses of MeHg can be dangerous if only the expression of MRP1 is reduced. Under such conditions, MeHg concentrations that are normally non-cytotoxic, may pose a considerable risk by increasing oxidative stress in the placenta promoting tissue damage. Therefore, genetic variants that reduce the MRP1 expression and impair its functionality (Szentpetery et al. 2004; Yin and Zhang 2011) could have detrimental consequences for the placenta and subsequently the child itself. The effects of *ABCC1* sequence variations on placental expression and functioning of MRP1 and the placental thiol redox status, however, are not known. It is therefore essential to investigate them. In the long run, our results could be integrated into clinical practice to early identify those children who may be particularly sensitive to exposure to MeHg (and other toxic MRP1 substrates) due to decreased MRP1 expression in their placenta.

### Localization of MRP1 in the human placenta

We evaluated previous publications on placental MRP1 localization and found either no experiment related to antibody validation (Kozlowska-Rup et al. 2014) prior to antibody application in IHC/IFM, different antibodies used in western blot and IHC experiments (Afrouzian et al. 2018) or the use of highly enriched membrane fractions in combination with a limited display of results in western blotting experiments (Atkinson et al. 2003; Nagashige et al. 2003; St-Pierre et al. 2000) (Suppl. Table 1).

MRP1 is widely expressed in normal tissues including placenta (Flens et al. 1994). While early studies reported mainly on MRP1 expression in epithelial cells (Flens et al. 1994), expression in endothelial cells (e.g. brain micro vessel endothelial cells) was later also confirmed (Calatozzolo et al. 2005; Mueller et al. 2005; Zhang et al. 2000). Using the validated antibody, we demonstrated by western blotting and IFM, expression of MRP1 in the STB and pFECs, with higher expression in pFECs. Expression of MRP1 in pFECs has been observed by most (Atkinson et al. 2003; Nagashige et al. 2003; St-Pierre et al. 2000), but not all studies (Afrouzian et al. 2018; Kozlowska-Rup et al. 2014) dealing with placental MRP1 expression. All studies demonstrated expression in the STB, except for St-Pierre et al. (2000), who had employed 2 different antibodies; one of them reacted with both, the STB and pFECs [anti-MRP1(m5)], while the other reacted only with pFECs [anti-MRP1(m6)]. As we showed expression of MRP1 in STB and pFEC not only by IFM but also western blotting, we are confident that both cell types express the protein. Due to the differences in expression level between STB and pFEC it is plausible that expression in the STB might not be detected when antibody dilutions are too high or exposure times during image acquisition are too low.

The mainly vesicular intracellular localization of MRP1 in STB and pFECs was puzzling given the fact that these drug transporters are generally considered to be cell surface localized and to mediate drug resistance by lowering total intracellular drug concentrations. Thus, also for placenta, ABC-type transporters are always discussed as being mainly plasma membrane localized (Dallmann et al. 2019). However, there are a variety of reports on intracellular localization of MRP1. Flens et al. (1996) tested an impressive number of healthy tissues and tumor tissue samples by IHC using three antibodies [anti-MRP1(m5) and anti-MRP1(m6) and MRP1r1]; these antibodies were later on also employed by St-Pierre et al. (2000) and Nagashige et al. (2003) for placental MRP1 localization. In healthy tissues, they always observed a cytoplasmic staining pattern, while in tumor tissues and in a cell line stably transfected with MRP1, plasma membrane localization was found. Wioland et al. (2000) also demonstrated intense cytoplasmic localization of MRP1 in various cells of the normal human nasal respiratory mucosa by using antibody MRP1r1. The predominant cytoplasmic expression of MRP1 observed in normal tissues suggests that the intracellular localization of MRP1 might have a physiological role. In response to unconjugated bilirubin exposure, for instance, MRP1 rapidly translocated from the Golgi to the plasma membrane suggesting that intracellular MRP1 could serve as a cellular reservoir (Gennuso et al. 2004). Intracellular MRP1 expression has also been demonstrated in various cell lines and the subcellular organelles with MRP1 accumulation were identified as endocytic vesicles, perinuclearly located lysosomes (Kim et al. 2015; Rajagopal and Simon 2003), trans-Golgi vesicles (Gennuso et al. 2004; Van Luyn et al. 1998) or mitochondria (Dartier et al. 2017; Jungsuwadee et al. 2009; Roundhill and Burchill 2012; Van Luyn et al. 1998). MRP1-dependent secretion of drugs into some of these organelles has been shown (Rajagopal and Simon 2003; Van Luyn et al. 1998); thus, MRP1 might allow for sequestering the drugs into intracellular compartments. Alternatively, MRP1 is a transporter of many endogenous substrates such as Vitamins or GSH/GSSG (Cole 2014). Transport of these endobiotics into subcellular organelles might be of relevance for their proper function or for storage purposes.

Rajagopal and Simon (2003) used transient instead of stable transfection of HeLa cells to induce MRP1 expression. When the plasmid was poorly expressed, the protein was found only in intracellular vesicles, and not at the plasma membrane at all. Intracellular localization (plasma membrane versus intracellular organelles) might thus depend on the level of protein expression resulting in more pronounced accumulation at the plasma membrane when expression is stimulated. Alternatively, it might also depend on the cell type under investigation. Expression of the same MRP1-cDNA containing plasmid in two different cell lines (HL-60 and HeLa cells) resulted in plasma membrane localization in one cell type (HeLa cells) and Golgi-localization in the other (HL-60) (Kaufmann et al. 2008).

In addition to the intracellular localization in the STB, we also observed some staining in the basal membrane of STB. These results are consistent with previous publications showing predominant expression of MRP1 in basal membranes of trophoblast cells (St-Pierre et al. 2000; Atkinson et al. 2003; Nagashige et al. 2003) supporting the assumption that MRP1 mediates the transport of MeHg from trophoblast cells towards the fetus. It is not known via which transport mechanisms MeHg enters pFECs. The localization of MRP1 at the luminal membrane of these cells indicates that the transporter is involved in the further transfer of MeHg into the fetal bloodstream. Because MRP1 is also localized at the abluminal membrane of pFECs, it could play a role in the transport of MeHg into the placental stroma and thus contribute to the accumulation of the metal in the human placenta (Gundacker and Hengstschlager 2012).

### The overall picture

**Fig. 8:**
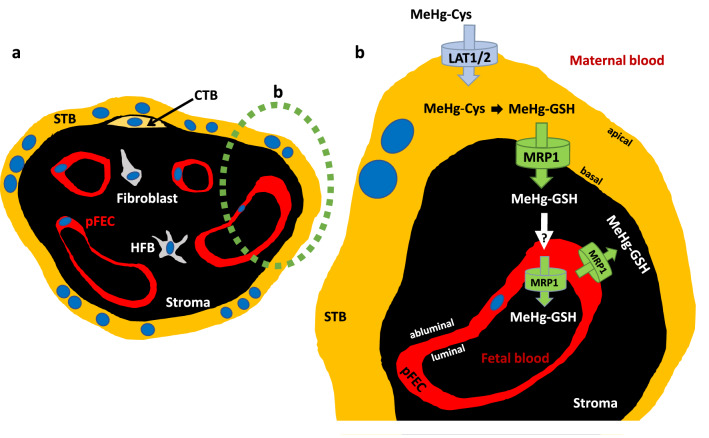
Model of MeHg transport across the placental barrier. **a** Schematic cross-section of a chorionic villus, as present in term placenta and **b** the placental barrier in detail. Here, the amino acid transporters LAT1 and LAT2, which are apically localized at the STB, are involved in MeHg-cysteine uptake in trophoblast cells. In the cell, MeHg dissociates from cysteine to bind to GSH. This MeHg-GSH conjugate is released by MRP1 that is expressed at the basal membrane of the STB. The subsequent mechanism and route of MeHg uptake in pFECs remains to be clarified. MRP1 is localized in pFECs, with expression at the luminal and abluminal plasma membrane, suggesting that MRP1 is involved in the transport of MeHg into fetal blood and possibly also into the placental stroma. References and further text are given in Chapter "The overall picture" (color figure online)

If we combine the results of the present work with the already existing data, the following picture emerges, which is summarized in Fig. 8.

Our previous data showed that System L amino acid transporters LAT1 and LAT2 localized at the apical (maternal side facing) membrane of the STB are involved in MeHg uptake into placental cell line BeWo (Balthasar et al. 2017) and primary human trophoblast cells (Straka et al. 2016). In a cell, MeHg dissociates from cysteine to preferentially bind to GSH (Farina and Aschner 2019; Rush et al. 2012). In this form, MeHg could be a substrate of efflux transporters such as MRP1 that are well-described to transport GSH-conjugates (Cole and Deeley 2006).

As we could demonstrate, MeHg actually is transported by MRP1. Together, the functional data of MDCKII cells overexpressing MRP1 (MRP1 is localized at the basal plasma membrane) and the basal expression of MRP1 in human STB (Figs. 6, 7) suggest that MRP1 is one of the most important efflux transporters of MeHg-GSH from STB. The mechanism and route of MeHg into the pFECs remain to be clarified (Fig. 8). According to our data, MRP1 is expressed in pFECs; occasional enrichment at the plasma membranes, specifically at the luminal membranes, was observed. It can therefore be assumed that MRP1 is involved in the transport of MeHg-GSH into the fetal blood and into the placental stroma. For simplicity, intracellular localization of MRP1 that we observed in our study is not displayed in Fig. 8. Further investigations are required to identify the type of organelle and the function of MRP1 in these subcellular structures.

According to our hypothesis (i.e. MRP1 knockdown in HTR-8/SVneo cells significantly reduces the efflux of MeHg-GSH and GSSG), downregulation of MRP1 led to increased MeHg accumulation and enhanced GSSG levels in the cells. What we did not expect were reduced cell numbers, reduced cell viability, increased apoptosis and oxidative stress even at the very low MeHg concentration of 0.03 µM (about 6 µg/L). However, such a low dose of MeHg is clearly non-cytotoxic when MRP1 is fully active.

## Conclusion

We show that MRP1 is essential for the proper function of trophoblast cells. It is required for export of MeHg to prevent cell death and also—in the presence of an oxidant such as MeHg—to export oxidized GSH to maintain a balanced redox status of the cells. Results from an epithelial cell line and the in situ localization suggest that MRP1 is crucial for the transfer of MeHg from the maternal circulation to the placenta and fetus.

## Electronic supplementary material

Below is the link to the electronic supplementary material.

Supplementary file1 (DOCX 292 kb)

## Data Availability

The manuscript together with supplementary information contains all data supporting the results of this study.
