# Supplementary material for: In vitro function and in situ localization of Multidrug Resistance-associated Protein (MRP)1 (ABCC1) suggest a protective role against methyl mercury-induced oxidative stress in the human placenta
Source: Arch Toxicol. 2020 Sep 11;94(11):3799–817. doi: 10.1007/s00204-020-02900-5 (PMC7603445; doi:10.1007/s00204-020-02900-5)
Supplement: Supplementary file 1 — Supplementary file1 (DOCX 292 kb) [file 204_2020_2900_MOESM1_ESM.docx]

**Supplemental Data**


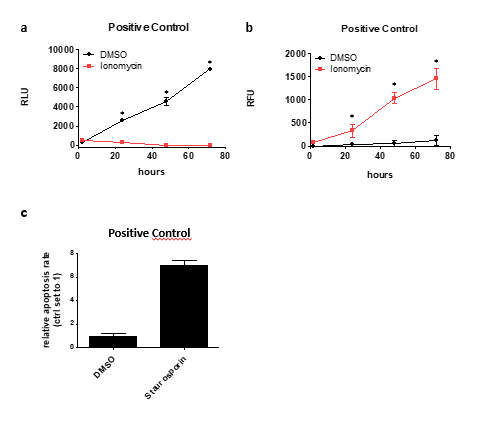


**Supplemental Figure 1**. Positive Controls for Viability **(a)**, Cytotoxicity **(b**) and Apoptosis **(c)**. Ionomycin (10 µM) or Staurosporin (1 µM) were used as positive control. The data represent mean values ± SD from three independent experiments. *P<0.05 from paired students t-test.


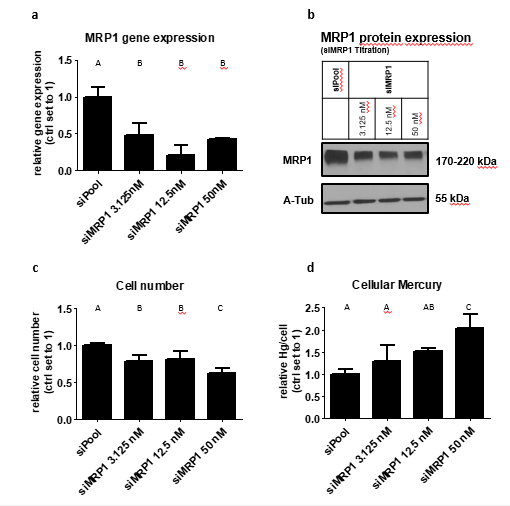


**Supplemental Figure 2.** Effect of MRP1 siRNA titration and treatment with 0.9 µM MeHg on HTR-8/SVneo cells. MRP1 knockdown is confirmed at **(a)** gene expression level and **(b)** protein level. **(c)** Cell number is significantly decreased after MRP1 knockdown and treatment with 0.9 µM MeHg for 14h. **(d)** Cellular mercury is increased by silencing MRP1 in a dose-dependent manner. The data represent mean values ± SD of three independent experiments. Letters A-C denote homogenous subgroups from one-way ANOVA and S-N-K post hoc test (P<0.05).


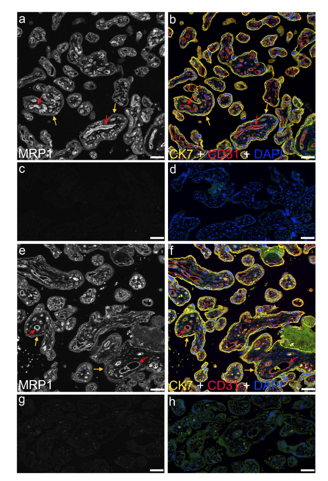


**Supplemental Figure 3. Localization** **of MRP1 in two third trimester placental chorionic tissue analyzed by multiplex staining and widefield fluorescence microscopy**. MRP1 expression (white in a and c) was observed in the STB (yellow arrows) and pFECs (red arrows) of all villi (a, e). STB and pFECs were identified by CK7 (yellow) and CD31 (red) expression, respectively (b, f). Nuclei were stained with DAPI (blue). Negative control sections of individual placentas, where primary antibodies were omitted, were processed under identical conditions (c, d, g, h). Within the STB, MRP1 staining appeared as vesicular pattern (yellow arrows) as well as membrane lining (yellow arrowsheads). Representative images of two out of five analyzed placentas are displayed. Images were taken with a 20x objective. Bars represent 50 µm.

**Supplemental Table 1**. Summary of previously used anti-MRP1 antibodies, mode of validation and observed placental MRP1 localization.

| **Type of pregnancy** | **Placental cell type showing expression by IHC/IFM** | **AB used for IHC/IFM Fixation** | **Localization in STB, pFEC by IHC/IFM**  ***By WB on placental MVM (apical) or BM (basal), AB & included controls*** | **Reference** |
| --- | --- | --- | --- | --- |
| Healthy  Healthy | pFECs only or STB < pFECs  STB and pFECs | MRPm5, Alexis (1:10)  MRPm6, Alexis  (1:10)  Frozen tissue/Formaldehyde  MRPr1, MONSAN  (n.a.)  Frozen tissue/Aceton | STB: apical  pFEC: diffuse  *WB: apical >> basal*  *MRPm5 + MRPm6, Alexis*  *Co+: HepG2*  STB: basal  pFEC: abluminal  *WB: basal >> apical*  MRPr1 (MONSAN)  Co+: LLC-MRP1, Co-: LLC-PK1/V | St. Pierre et al.,  2000  Nagashige et al., 2003 |
| Healthy | STB<pFECs | MRPm6, Chemicon  (1:20)  Formaldehyde | STB: diffuse staining (cytoplasm?)  pFEC: diffuse staining  *WB: basal >>> apical*  *MRPm6, Chemicon +*  *MRPr1 (Alexis)*  *Co: n.a.* | Atkinson,  2003 |
| Healthy  GDM | STB (not pFECs+)  STB🡻 | QCRL-1, Santa Cruz  (1:500)  Formalin | STB: basal >>>apical  *WB: n.a*. | Kozlowska-Rup et al., 2016 |
| Healthy  PE | STB >>>pFECs  STB🡹>>>pFECs  (PE: mRNA MPR1🡹) | ab24102, Abcam  (1:160)  Formalin | STB: apical>cytoplasm  STB: cytoplasm🡹  *WB: basal >> apical*  *Using QCRL-1, sc-18835, Santa Cruz*  *Co+: MRP1-baculovirus infected insect cells (Sf9)*  *Co-: Sf9* | Afrouzian et al., 2018 |

Abbreviations: AB: antibody; BM: basal plasma membranes; Co+: positive control; Co-: negative control; GDM: gestational diabetes mellitus; pFEC: human placental fetal endothelial cells; IFM: Immunofluorescence microscopy; IHC: Immunohistochemistry; LLC-PK1: porcine kidney epithelial cell line; MVM: Microvillous plasma membranes; n.a.: not applicable; PE: preeclampsia; STB: syncytiotrophoblast; WB: Western Blot.
